# Technology opportunity discovery by structuring user needs based on natural language processing and machine learning

**DOI:** 10.1371/journal.pone.0223404

**Published:** 2019-10-29

**Authors:** Taeyeoun Roh, Yujin Jeong, Hyejin Jang, Byungun Yoon

**Affiliations:** Department of Industrial & Systems Engineering, School of Engineering, Dongguk University, Seoul, South Korea; Fayoum University Faculty of Computers and Information, EGYPT

## Abstract

Discovering technology opportunities from the opinion of users can promote successful technological development by satisfying the needs of users. However, although previous approaches using opinion mining only have classified various needs of users into positive or negative categories, they cannot derive the main reasons for their opinion. To solve this problem, this research proposes an approach to exploring technology opportunity by structuring user needs with a concept of opinion trigger of objects and functions of the technology-based products. To discover technology opportunity, first, an opinion trigger is identified from review data using Naïve Base classifier and natural language processing. Second, the opinion triggers and patent keywords that have a similar meaning in context are clustered to discover the needs of the user and need-related technology. Then, the sentimental values of needs are calculated through graph-based semi-supervised learning. Finally, the needs of the user are classified in resolving the problem of vacant technology to discover technology opportunity. Then, an R&D strategy of each opportunity is suggested based on opinion triggers, patent keywords, and their property. Based on the concept of opinion trigger-based methodology, a case study is conducted on automobile—related reviews, extracting the customer needs and presenting important R&D projects such as an extracted need (cargo transportation) and its R&D strategy (resolving contradiction). The proposed approach can analyze the needs of user at a functional level to discover new technology opportunities.

## Introduction

Technology innovation has accelerated the growth of the economy by enabling the development of new products and services. Thus, discovering new technology opportunities can be regarded as a fundamental activity of government, as well as companies. Basically, since two opposite approaches, such as technology push and demand pull, are the main sources of innovation, most traditional studies on technology forecasting started their arguments by choosing their position in two approaches. While technology push means that a new technology catalyzes impactful innovation by utilizing and commercializing the technology, the demand-pull approach refers to needs of users for a product or technology that brings in innovation to satisfy their needs. Conventional studies on technology innovation highlight the role of technology push, because a technological breakthrough is regarded as the only driving force for innovation. However, other researchers advocating a demand-pull approach concentrate on user needs, investigating a diverse set of market features [1]. While users do not always need a high technology product, they just want to satisfy their needs at a reasonable price and performance. Thus, it is suggested that user-oriented experiences and designs are a leading factor in the competitive marketing environment [2]. In addition, previous studies proposed an R&D framework that reflects users’ needs through opinion mining [3, 4].

However, there are two limitations to the previous studies about TOD using opinion mining. First, they just profile the user, or classify their opinion. In other words, they do not focus on discovering “what they want” and “how it works” in a structured manner. Although classified opinions can provide the information on how a user thinks about their products and services, existing approaches cannot suggest a way to satisfy users by developing new technology, or by modifying current technology. Second, the characteristics of technology and user needs are not directly connected. Since patent assignees might be experts in specific technology, they write a patent script in their technical terminology. In contrast, reviews of users are written in simple words, because users do not have scientific knowledge. Due to these differences, user opinions can hardly be reflected in the R&D process, even when they express their opinions with objective information in words. Thus, previous researches are limited to a specific technology area in which only the pre-defined area can be analyzed.

Since previous studies on opinion mining concentrate on classifying the opinions of users into positive or negative categories, they cannot derive the main reasons for their opinions. Thus, they cannot be actively utilized to suggest new technology opportunity to satisfy user needs. To solve this problem, this paper defines an opinion trigger as an object that users express their feeling towards products or services. In other words, an opinion trigger means the main reason for user opinion. The opinion trigger can be interpreted as “what the user wants, or “how it works”. Furthermore, user needs can be constructed by the opinion trigger. In this research, each component of product/technology is discovered as needs that a user wants to be satisfied. The property of needs and related technology can be used to build a technology strategy to satisfy user needs in designing products and services. In addition, the word-embedded matrix is used to find a relationship between user needs and technology information. Based on their relationship, the words that express differently but have the same meaning can be derived. Thus, an R&D process can be efficiently managed, because the proposed approach can reduce a pre-defining process, or expert advice.

This paper is organized as follows. Section 2 presents the theoretical background, which contains technology opportunity discovery (TOD), which is the main purpose of this research, opinion mining analyzing the needs of the user, and technology backgrounds, which are natural language processing and Word2Vec. Section 3 presents the research concept and framework, and explains the details of the research process. Section 4 conducts a case study in the car industry, to illustrate the proposed approach. Finally, Section 5 discusses the contribution of this research, its limitations, and the possibility of future research.

## Theoretical background

### Technology opportunity discovery (TOD)

The purpose of technology opportunity discovery (TOD) is to identify significant trends in society, culture, and science to discover the most promising technology [5]. Technology opportunity includes the possibility that may be applied in various industries, not confined within a specific industry [6]. Previous TOD approaches based on patents and papers use bibliographic information and their text data. Since patent information contains a variety of technology information, important properties of a technology field can be extracted by patent mining. Promising technology fields are identified [7], while the relationship between various technologies using a citation network is derived [8]. Technology trends are analyzed based on the number of registered patents each year [9, 10], while IPC code is used to find the core technology in an environmental ecology field [11]. RFID technology is forecasted in China through clustering and patent portfolios [12], while exploring promising technology sectors by using patent networks and promising patent index [13].

In addition, various text mining techniques and statistical analysis are employed to discovery emerging or promising technology fields. Text mining, which is the main method of extracting significant information from patent qualitative data, is highly dependent on keyword extraction. To increase text mining performance and prediction accuracy, many methodologies, such as Latent Semantic Analysis and Latent Dirichlet allocation, have been proposed. Core keywords are identified in the parts of summary and claims, then identify the targets of mergers and acquisitions or patent infringements [14, 15]. Technology development is analyzed in shale gas development in China and the United States, using the abstracts of patent documents [16]. In addition, novel patents are find to predict technology convergence based on keyword similarity [17]. In particular, technology opportunity is identified through subject-action-object (SAO) text mining and morphology analysis [18]. Product features that customers want are grouped by using LDA [19], while opinion mining and clustering is conducted to characterize products [20]. In addition, product ranks and their features are suggested based on opinions and the interests of users [21].

The TOD, which has the perspective of technology push, can obtain competitive priority based on high technology ability [22]. However, innovative or high-performance technology does not always get competitive priority from a customer choice. Some technology or product has higher technology ability than is necessary, and the price is too expensive to pay for it. In addition, R&D activities without market prediction may result in useless technology for users. Furthermore, the development of social network services and Internet shopping services allows users to easily express and share their opinion. Based on other user opinions, they can choose the products and services that they truly want, forcing technologists to reflect their opinions in developing a product, and even a technology.

### Opinion mining

Opinion mining extracts quantitative and qualitative data from user opinion data through natural language processing (NLP), linguistics, and text mining [23]. The technique is also called sentimental analysis, by deriving positive, neutral, and negative opinion. This drives sentimental value, meaning the quantitative value of opinion. A positive value means a positive opinion about an object, while a negative value means a negative opinion about an object. Opinion mining can be divided into sentimental analysis and contents analysis. Sentimental analysis derives sentimental value from sentences or documents. Pre-defined opinion dictionaries, such as Semantria and SentiWordNet, are used as an opinion dictionary for sentimental analysis. Well-expressed opinions are extracted by analyzing movie review data [24]. Sentimental value prediction models are developed through analyzing opinion text data and user profiles, such as age, sex, and religion [3]. Technology opportunities are discovered by analyzing user reviews and patents through sLDA [25]. Opinion mining is used to quantify the degree of positive or negative opinions about products or services [4, 26]. Also, posts on social network service such like Twitter are used for opinion mining to identify and monitor the development trends of emerging technologies including patent analysis [27]. Not only user opinions are used to form text; various factors are used together to improve the reliability of analysis. Complaints are analyzed by using reviews containing a grade [3], and user information, such as age and gender, is used to predict user needs [28]. Using machine learning algorithm, many studies to improve accuracy and extract more useful information have been conducted. Extracting multiple linguistic features is suggested in an automatic learning system to summarize many reviews [29]. Deep learning models are applied such as recurrent neural network and convolutional neural network, for more accurate sentimental analysis and features of opinions [30–32].

In the R&D process, the needs of users are identified through opinion mining, and can be reflected in technology attributes, or specification to satisfy users. User reviews are analyzed with their profile and predict future users [28], while needs of users are extracted by analyzing relationships with each word, sentence structure, and specific words occurrence [33, 34].

### Natural language processing (NLP)

NLP is linguistics based on computer science that studies the interaction between computer and human. Its theoretical base resides in supervised machine learning. NLP machines learn from the linguistic information of each sentence or document, such as phrase, part-of-speech (POS), and sentence structures, and analyze new input sentences or documents based on learned information, and derive linguistic information. NLP can extract more linguistic information from texts than simply using the meaning of each text. Its function can be divided into chunking and POS tagging. Chunking is also known as parsing, extracting phrases expressing one object with other words. POS tagging is tagging a part-of-speech, which means the linguistic function of each word in a sentence.

Chunking is conducted in an automatic translation area. A translation algorithm is suggested by dividing complex sentences into single sentences, and chunking various sentences, then translating texts at a phrase level [35]. The effectiveness of the phrase level translation machine is proved by comparing with word level or sentence level translation [36, 37]. POS tagging is used in many areas that need more detailed information about text. Text mining is divided into keywords-based and key function-based approaches [38]. Nouns and verbs of each sentence are extracted from the perspectives of subject-action-object (SAO), and collected patents are analyzed based on SAO structure. Nouns and verbs are interpreted as functions and objects, and then construct a technology tree based on a patent’s function and object [39]. An inventive design method is extracted, based on TRIZ and patent semantic similarity [40]. Furthermore, technology information is structured in patent documents, using their linguistic character and patent law [41].

In this research, chunking is used to divide a complicated sentence into single sentences. Then, POS-tagging is used to identify components of needs structure. Users express their opinion in the adverbial and adjectival forms, thus these words are defined as sentimental keywords. Using sentimental keywords, sentences from user opinion are classified into opinion sentences and non-opinion sentences. In addition, opinion triggers are classified into object-related opinion triggers and function-related opinion triggers based on their POSs, which are verb or noun.

### Word2Vec

Word2vec is a word embedding method that can rapidly calculate more than a billion words [42]. Word embedding is a methodology to represent texts in a vector space by reducing the dimensions of the text vector that form its own vector space. Many embedding models have been suggested based on the neural network; feedforward neural net language [43], and Recurrent Neural Net language Model [44]. Word2vec uses a continuous bag of words (CBOW) and a skip-gram learning model. CBOW predicts which word appears next to or between texts, when various words appear through learning. Skip-gram predicts words that appear next to previously appearing words through learning. Using these learning models, Word2Vec has the advantage of word embedding that learns a lot of data into a vector space with a short learning time, compared to other word embedding methodology.

Word2Vec has two specific functions. One is semantic similarity being represented in a vector space, and the other is a vector calculation that can be done between words in a vector space. Semantic similarity reflects the synonyms and derivatives of a word. Using the property of word occurrence representing their semantic similarity, similar words can be identified in a vector space without a predefined dictionary, such as Wordnet. Based on this character of Word2Vec, user opinion is classified using clustering methodology [45].

**Fig 1:**
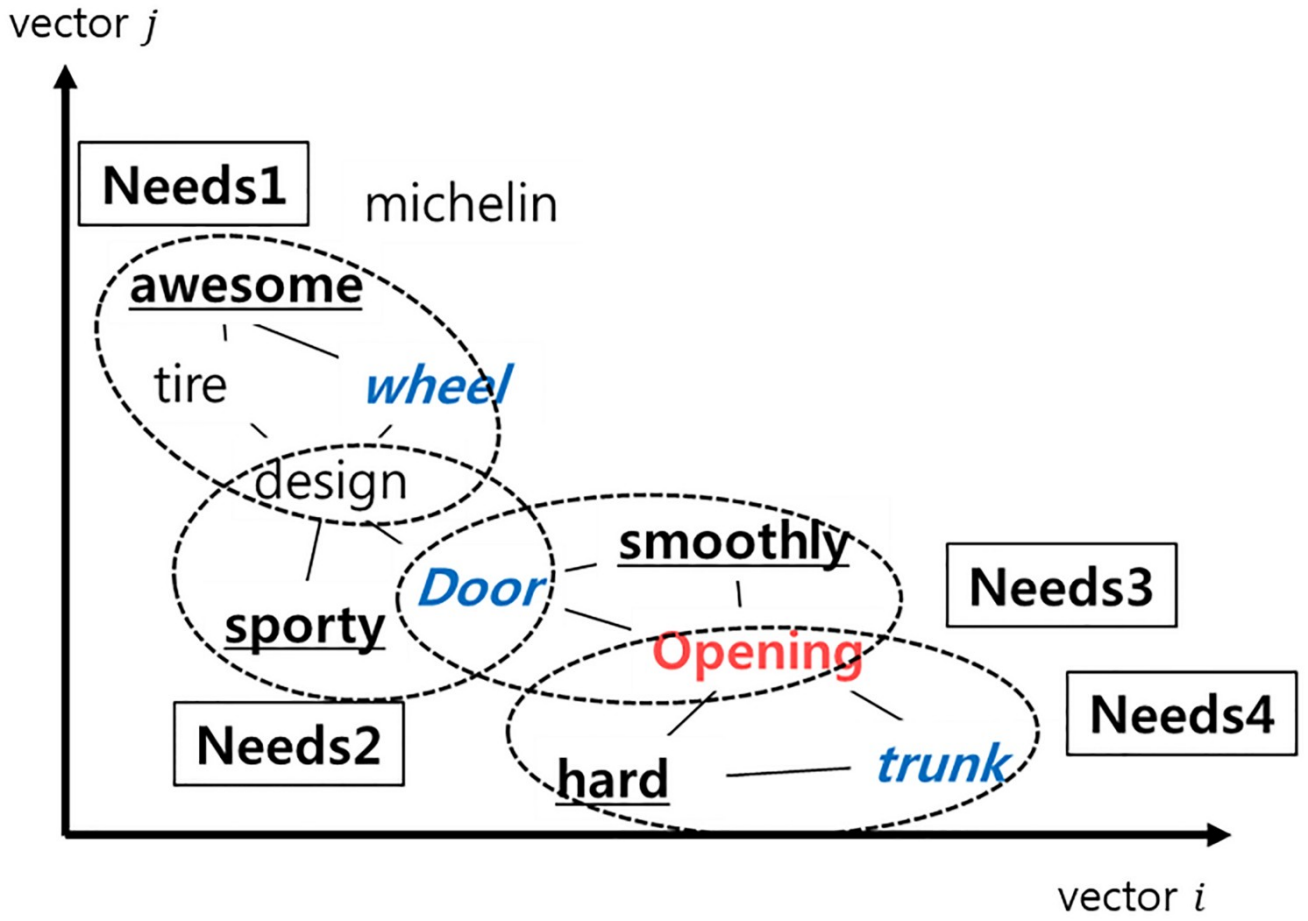
Concept of identifying needs in vector space. Underlined word, blue color word and red colored word expressing sentiment, object and function, respectively.

In this research, a combination of the words is identified as needs, using features that words having semantic similarity occur nearby. Each word having semantic similarity can be clustered using clustering methodology, and interpreted as needs. Patent and user opinions are embedded in the same vector space to word gap between expert and users. Fig 1 shows concepts of identifying the needs of user by Word2vec and clustering methodology.

## Methodology

### Research framework

**Fig 2:**
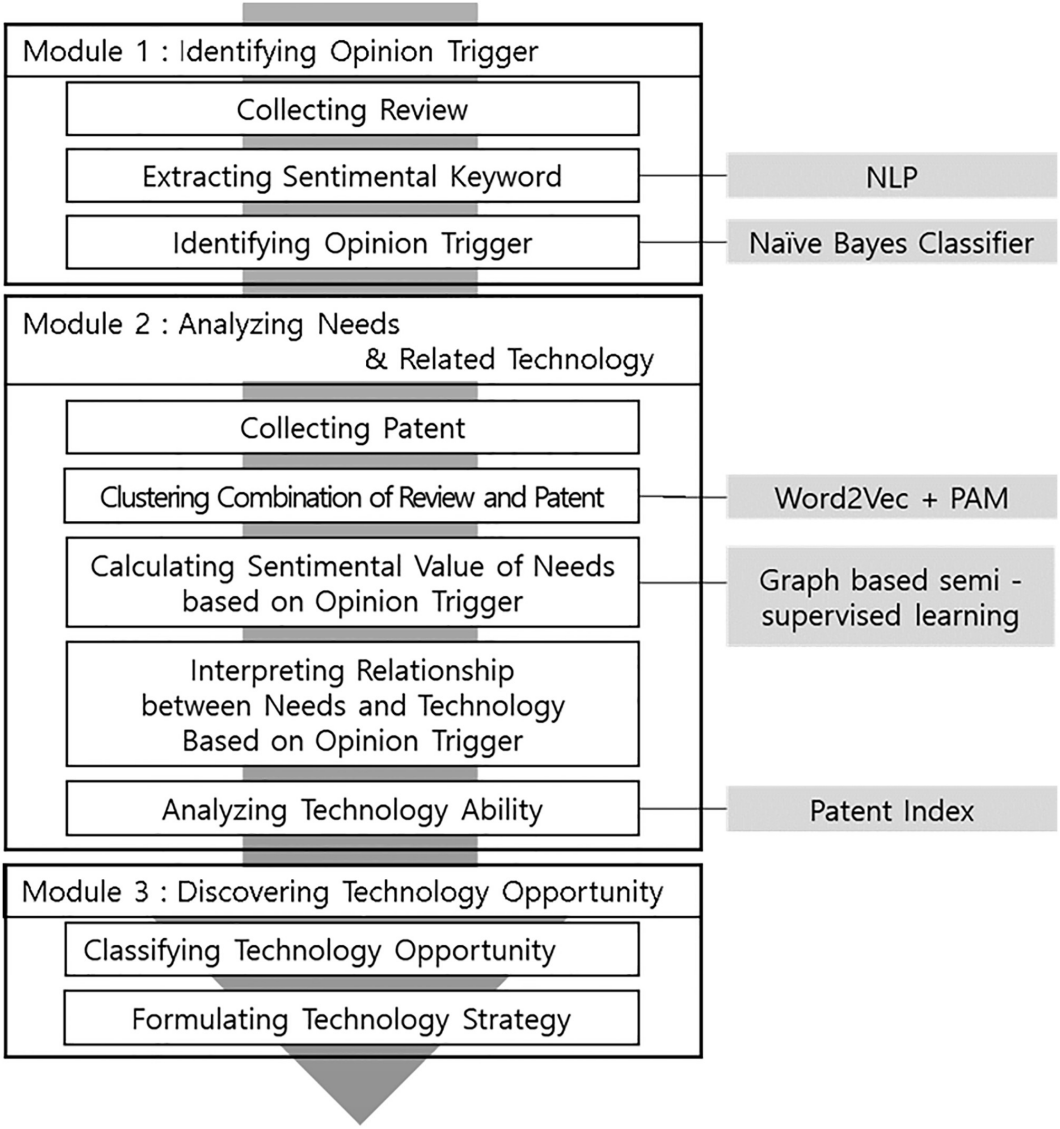
Research framework. Module construction (outer flow chart), detailed processes for each module (inner flow chart), and methodologies (tagged by right side boxes).

To discover technology opportunity based on opinion triggers, a framework that consists of three modules is suggested. In the first module, an opinion trigger is identified as an object or a function opinion trigger by Naïve Bayes classifier to detect an ‘opinion sentence’, which contains one of the sentimental keywords among all review sentences. The second module constructs the structure of user needs for each type of opinion trigger from both user needs and related technology points of view. Needs are analyzed by clustering combinational text data on both user reviews and patents through word2vec and partitioning around medoids (PAM). Sentimental value for each need is calculated by graph-based semi-supervised learning. Then the relationship between the needs and technologies is interpreted based on the structure of the opinion trigger and additionally, the technology ability is analyzed. The last module of discovering technology opportunity makes suggestions of technology opportunity types and technology strategies. Fig 2 shows the framework of this study.

### Detailed process of framework

#### Module 1: Identifying opinion trigger

In Module 1, the opinion trigger is identified as the source of needs. Despite the user expressing their opinion using various words, these opinions have the same linguistic characteristics. Those characteristics have the same linguistic structure—adjective or adverb + verb + noun. Based on the characteristics, opinion triggers are identified using natural language processing and naïve Bayes classifier. The POS-tagging function can be used to derive each part of speech in an opinion. POS-tagged words can be suggested as candidates of opinion triggers. In addition, naïve Bayes classifiers suggest the probability of each word usage when users express their opinions, and this probability is used to identify opinion triggers.

The first step of identifying opinion trigger is to crawl review data. Review data on a website express various user opinions. To identify opinion triggers, review data are collected from several websites that collect and show expert review data for users. Expert reviews consist of multiple sentences and detailed information about products. Since their purposes are to re-provide information for users, they have to explain detailed information, such as components, modules, and attributes of products in simple words that users can readily understand. Thus, it can be the best source of opinion mining and word2vec. The review data are collected on several websites, not on a specific website. Since some websites might have biased reviews about user propensities or specific products/companies, review data in websites are crawled by web crawling. This research uses a web crawler based on R using “httr”, “rvest”, and the “RSelenium” package.

In the review data, there is much information beyond the reviewer’s opinion about product/technology. For example, the data could contain product specifications or their purpose of purchasing the product. This information does not express an opinion. A difference between such information and user opinion is the occurrence of adjectives or adverbs. In the case of user opinion, users express their feeling by using adjectival or adverbial phrases, such as “great design!”, “the best wheel”, and “handles turns smoothly”. Since this research focuses on investigating these linguistic characteristics of user opinion, sentimental keywords are defined as words that are used when users express their opinion using natural language processing.

As mentioned above, sentimental keywords are formed as adjectives or adverbs. Based on natural language processing, candidates of sentimental keywords can be extracted. Then, the sentimental keywords can be classified based on their usage. For example, ‘good’, ‘best’, and ‘bad’ can be clearly interpreted by being combined with an object like examples such as “bad cargo!”, “bad handle” and “bad efficiency”. Thus, it can be defined as a global sentimental keyword (GSK), meaning a keyword that can express their sentiment in a global situation. On the other hand, words such as ‘smoothly’, ‘wide’, and ‘immediately’ cannot be significantly interpreted, when they are used with an object. For example, while the “stop immediately” is a reasonable opinion, the “immediately cargo” cannot be interpreted and does not a reasonable opinion. The word “stop” has the word “immediately” as an attribute like “quick” and “fast”, whereas the “cargo” does not. Therefore, “immediately cargo” cannot be interpreted. Thus, these words that can be interpreted with a specific object are defined as a static sentimental keyword (SSK), meaning a keyword that can express their sentiment in a static environment. The two types of sentimental keywords have the same meaning as a global variable and static variable in a programming language. Sentimental keywords are used to identify opinion triggers, and the two types of sentimental keywords are used to calculate the sentimental value of needs based on their attribute.

An opinion trigger is defined as words that users use to express their opinion directly. Based on the definition of a sentimental keyword, the opinion sentence has to include sentimental keywords. Using this character of user opinion, an opinion trigger can be identified from user opinion, as follows. First, user opinions that consist of several long sentences in review data are partitioned into multiple single sentences. Second, each sentence is classified into opinion sentence and non-opinion sentence, based on the occurrence of sentimental keywords in the sentence. If a sentence has a sentimental keyword, this means that the sentence can be classified as user opinion. On the other hand, if not, the sentence can be classified as a non-opinion sentence. This means that there is no opinion in the sentence. Third, opinion triggers are identified using naïve Bayes classifier.

Naïve Bayes classifier is a supervised machine learning methodology based on the assumption that each probability distribution is independent of each other. Bayes theory, which is a theoretical base of Naïve Bayes classifier is used to calculate the conditional probability that is hard to calculate using other conditional probability. Eq (1) shows the basic formula of Bayes theory:

$$P(A|B)=\frac{P(B|A)\times P(A)}{P(B)}=\frac{P(A\cap B)}{P(B)}$$ (1)

According to the definition of opinion trigger, the words used when users express their opinions can be an opinion trigger. It can be defined as P(Words | Opinion Sentence). In Bayes formula, P(Words | Opinion Sentence) and P(Words | Non-opinion Sentence) could be obtained using Eqs (2) and (3). If a word has a higher P(Word | Opinion sentence) than P(Word | Non-opinion Sentence), the word can be identified as an opinion trigger. This means that the word occurs more frequently in opinion sentences than non-opinion sentences. Logically, sentimental keywords always have high probability. However, sentimental keywords do not contain “what they want” or “how it works”. Thus, except sentimental keywords, the keyword that has high P(Word |Opinion sentence) can be identified as an opinion trigger.

$$P(Word|Opinion\;Sentence)=\frac{P(Opinion\;sentence|Word)\times P(Word)}{P(Opinion\;sentence)}$$ (2)

$$P(Word|Non-opinion\;Sentence)=\frac{P(Non-opinion\;sentence|Word)\times P(Word)}{P(Non-opinion\;sentence)}$$ (3)

Fourth, the identified opinion triggers are classified into function opinion trigger (FOT) and object opinion trigger (OOT). As mentioned above, the needs of users can be constructed with adjective/adverb, verb, and noun. Since adjective or adverb are defined as a sentimental keyword, verb and noun should be defined to construct user opinions. As an opinion trigger is defined using the probability of each word, POS-tagging in natural language processing can be used to define the verb and noun in opinions. Thus, an opinion trigger formed as a verb can be defined as FOT, while an opinion trigger formed as a noun can be defined as OOT.

#### Module 2: Identifying needs and related technology

In Module 2, needs and related technology are identified based on opinion triggers using Word2Vec and PAM. Then, the sentimental values of needs and technology ability are analyzed by using graph-based semi-supervised learning and patent index. Sentimental value means how a user is satisfied by each need, and the related technology ability means the ability to satisfy the needs of a user. Based on these properties, technology opportunity is classified in Module 3.

At first, patents are collected to analyze technology information and their technology ability in the United States Patent and Trademark Office (USPTO) database. Patents that are issued in the USPTO are approved by patent law, and have technology information in a clearly explained sentence, with no grammatical or linguistic errors [46]. Thus, review data and patents could be a source of analysis, reducing the words-gap in the same vector by reflecting the opinions of users and technology information. To collect patent documents, a query is used that restricts time period and the scope of industry. On average, a patent takes two years from application to issue. Thus, this research analyzes patents applied from two years ago for 10 years. To collect patents in a specific industry, this research uses simple keywords that represent the industry. Since specialized keywords, such as component or module, cannot drive the technology information about an industry, they are excluded in this research.

Needs of users could be discovered through a combination of opinion triggers and sentimental keywords. The basic formula of the needs of the user can be defined as OOT + FOT + (sentimental keywords). For example, in “handle turns smoothly”, “handle” is the main object of needs, “turns” is a function of how the handle works, and “smoothly” expresses the sentiment that the user feels. Even if there is no sentimental keyword, two types of opinion trigger can structure the user needs itself. “Gear downshift” can be an example of user need that is structured without a sentimental keyword. Also, several needs can be discovered by a modified formula: OOT / FOT + sentimental keyword. “Great downshift!” and “wide cargo space” can identify needs about a downshift and cargo. Each need can be examples of FOT (downshift) + sentimental keyword (great) and OOT (cargo space) + sentimental keyword (wide).

To find their combination of words, Word2vec and PAM are conducted. Word2vec is used to analyze the relationship between words based on review data and patent data, and then suggest the vector source of each keyword as their relationship. Based on these vector sources, PAM is conducted to cluster opinion triggers and sentimental keywords to discover the needs of the user. PAM, also known as k-medoids clustering, is a clustering methodology based on nodes. The center nodes of a cluster are called medoids, and the number of medoids can be selected by several criteria. PAM is more robust from outliers than k-means clustering. Furthermore, besides the number of medoids, each medoid that is a starting point to calculate in an iteration that can be selected by the analyst. It can make an opinion trigger as a center of the cluster, enabling an opinion trigger to be derived—a weighted cluster. This research conducts PAM, using the same number of clusters as opinion triggers to analyze the most related keywords in a vector space.

**Fig 3:**
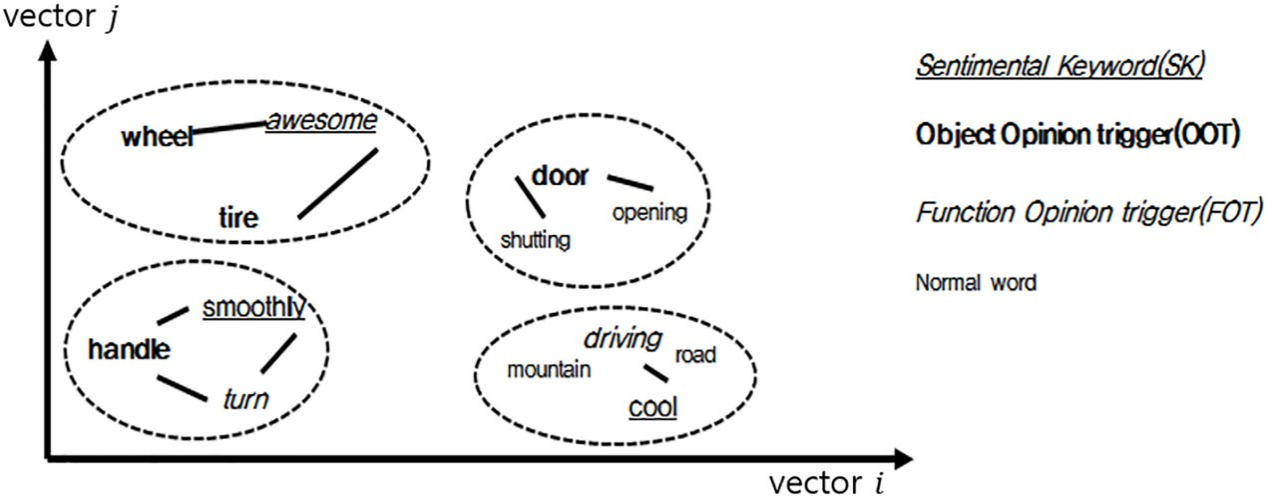
Identifying needs using combination of trigger (example). Underlined word, bold word and italics word expressing sentiment, object and function, respectively.

Fig 3 and Table 1 show an example of word2vec and PAM results. In Fig 3, several types of words are embedded in a vector space. The distance of each word means the degree of meaningful relationship, and in this case, four clusters are constructed by PAM. Needs #1 and #2 are formed by the basic formula, and they are constructed by OOT and FOT. Although need #2 does not have a sentimental keyword, the need can be discovered based on OOT and FOT. Need #2 has two FOTs expressed by different words; however, they have the same meaning. Thus, they can be suggested as the same needs. Needs #3 and #4 are formed by the modified formula, discovered by only one type of need. Although Need #3 has two different object triggers, they have the same meaning. Need #4 has normal keywords that are neither a sentimental keyword, nor an opinion trigger. These keywords that come from the opinions of users or patents can be used to analyze detailed information of needs. For instance, need no 4. has “mountain” and “road” as normal keywords and it can be useful information to discover detailed needs such as “driving in mountain or road”.

**Table 1:** Identifying needs using needs formula.

Needs No.	Needs formula	Trigger		Sentimental Keyword	Normal word	Needs
		Object	Function			
1	Basic Formula	Handle	Turn	smoothly	-	Turning of handle
2		Door	Shutting	-	-	Shutting(closing) door
			Closing			
3	Modified formula	Wheel		awesome	-	Wheel (Tire)
		Tire				
4			Driving	cool	mountain, road	Driving in mountain or road

The sentimental value of each need is calculated by using the property of the sentimental keyword. The sentimental value of needs can be derived from the sentimental value of the opinion trigger. The sentimental keyword is a word by which users express their feeling, and each keyword has sentimental value. However, an opinion trigger does not have its own sentimental value. To solve the problem, this research uses a structure of needs that is constructed with sentimental keywords and opinion triggers. That is, users express their opinions using sentimental keywords and opinion triggers together. Thus, each opinion trigger can have sentimental value based on co-occurrence with positive or negative sentimental keywords.

To calculate the sentimental value of the opinion trigger, the sentimental value of the sentimental keyword is propagated to each opinion trigger that forms user needs. For this, a graph-based semi-supervised learning technique that is one of the network analysis methodologies is employed. The specific value of one node is propagated to neighboring nodes, and it can derive the missing or empty value of the other nodes. In this research, the graph-based semi-supervised learning that is mainly used for network analysis is modified to apply to clustering analysis. Several types of keywords, such as opinion triggers and sentimental keywords, are embedded in a vector space, and clustered based on their relativeness. This means each keyword in the same cluster has a meaningful relationship. Thus, the graph-based semi-supervised learning can be conducted in cluster analysis, because each cluster can substitute a network as a group of a meaningful relationship.

**Fig 4:**
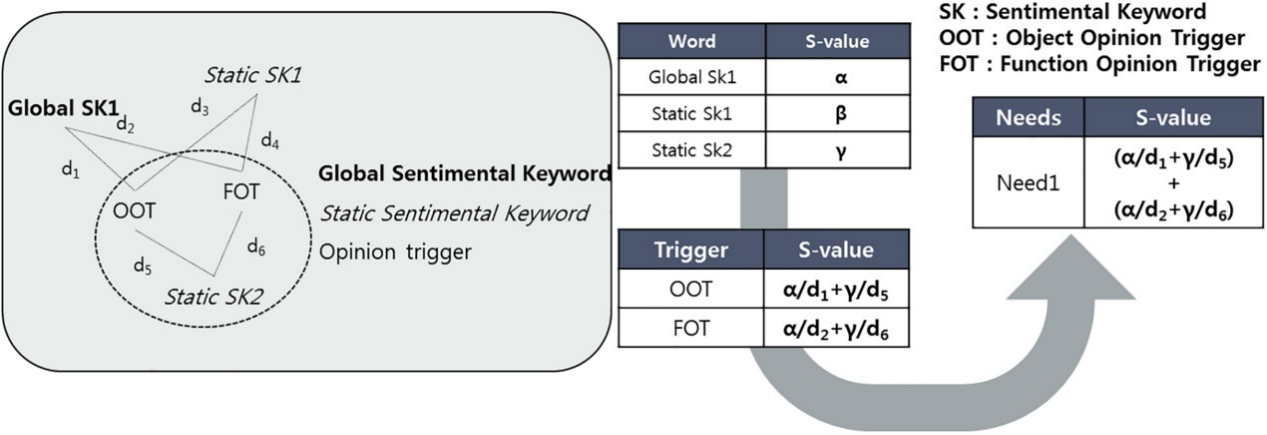
Process to calculate sentimental value of needs. A conceptual example of needs with SK and OT (left box) and flow chart of calculating s-value based on SK and OT (right flow chart).

Based on the graph-based semi-supervised learning, two types of sentimental keywords are propagated to the opinion trigger in different ways. First, the sentimental value of each sentimental keyword is calculated using Semantria. Semantria analyzes the sentimental value of documents, sentences, or words based on a pre-defined sentimental dictionary and their own algorithm. Many opinion minings have been conducted to calculate the sentimental value based on Semantria [25, 47]. Second, the distances between opinion triggers and sentimental keywords are calculated depending on the sentimental keywords type. SSK can express their opinion about a restricted opinion trigger. Thus, if an SSK does not belong to a cluster, its distance to an opinion trigger equals infinity. This means there is no relationship between the opinion trigger and the sentimental keyword. The GSK can express its opinion about all the opinion triggers, and its distance to an opinion trigger can be used in calculating sentimental keywords as a weight. The third step is to multiply the sentimental value of sentimental keywords by the inverse distance opinion trigger and sentimental keywords, to calculate the sentimental value of the opinion trigger using Eq (4). Since the distance between two types of words means their semantic similarity, it can be used as a weight in graph-based semi-supervised learning. Finally, the sentimental value of needs can be derived from the sum of the sentimental value of the opinion trigger using Eq (5). Fig 4 shows the process for calculating the sentimental value of needs.

$$Sentimental\;value({Opinion\;trigger}_{n})=\sum_{i=1}^{n}\frac{Sentimental\;value\;of\;{Sentimental\;keyword}_{i}}{Distance\;({Sentimental\;keyword}_{i},\;{Opinion\;trigger}_{n})\;}$$ (4)

$$Sentimetal\;value(needs)=\sum_{n=1}^{k}Sentimental\;Value({Opinion\;trigger}_{n})$$ (5)

**Fig 5:**
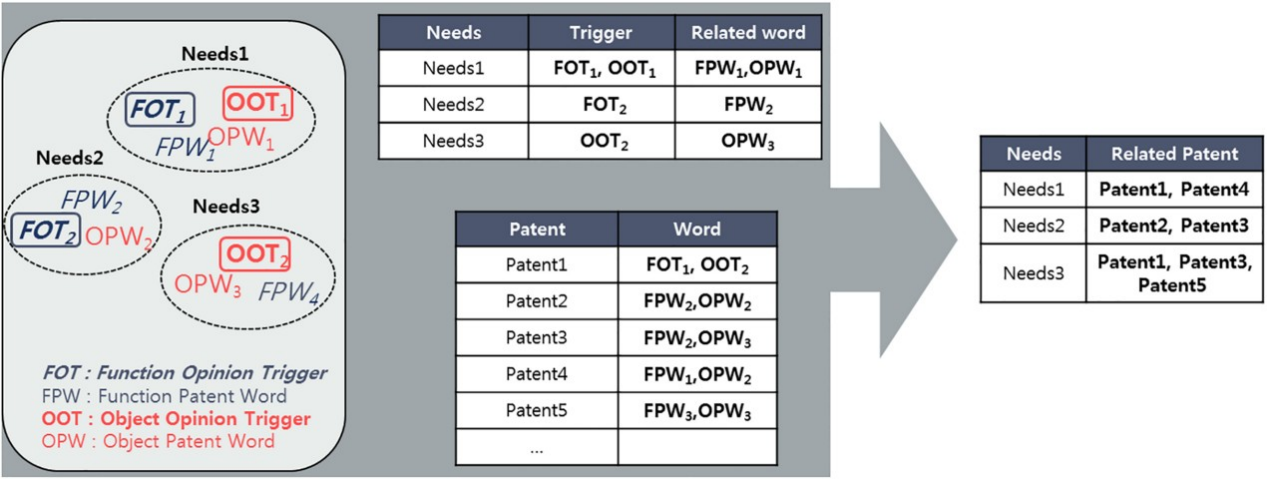
Process of identify needs-related technology. A conceptual example of needs with OT and related words (left rounded box), needs with trigger and related word (middle top table), patent list with related OT word lists (middle bottom table) and need list with related patents (right table).

User needs related technology is identified based on patent keywords in a cluster formed by needs. Using the POS-tagging function in NLP, keywords in patent documents are classified into noun, verb, and others. In this research, noun and verb words are suggested to be object patent words (OPWs) and function patent words (FPWs). Each word can be interpreted by patent object and patent function. Using these two types of patent words, user needs and related technology can be identified. Based on the opinion trigger structure of needs, related patent words are allocated in each need. In the case of need #1 that has both OOT and FOT, FPW and OPW in the same cluster can be interpreted to mean that the FPW and OPW have a relationship with need #1. Hence, patents containing those FPWs and OPWs are allocated in needs-related patents. Then, the group of patents can be suggested as needs-related technology. Fig 5 shows the process of identifying needs related technology.

The technological ability of needs-related technology can be analyzed using patent index. Patents have their own quantitative value, such as the number of cited patents, the number of citing patents and the number of independent claims, and the number of INPADOC family patents. These indices can be interpreted by their own meaning. The number of cited patents means how many other patents cite the patent to apply for a new patent, indicating that their technology information is used by other patents. Thus, the number of cited patents can be interpreted as the impact on other patents. The number of citing index means how many other patents are cited to apply for their patent. Thus, a high number in the citing index means that the novelty of patents is low, showing the number of citing patents can be interpreted as innovativeness. The claim is a right to legal protection of their novel invention from others. Many claims of a patent mean that it has a lot of technology information that can be protected by patent law. The INPADOC family number is their legal right in other countries. Once a patent has been issued in a country, its right can be protected in other countries included in the INPADOC family. Thus, the INPADOC family number can be interpreted as the marketability of the patent.

These patent indices show capability from various perspectives [13, 25, 48] impact, innovativeness, authorization, and market ability. Thus, the average value of the indices can be the technology ability containing various perspectives. Table 2 shows each formula of index and their meanings. Since each index has other meanings about technology property, the average of the four indexes is suggested as the technological ability. Then, the difference between the ability of each index and average index of the whole technology is derived as the technology ability of each needs-related technology.

**Table 2:** Meaning of patent index and formula.

Index	Formula	Meaning
Impact	$\frac{Avg.\;Cited\;Num-min(Avg.\;Cited\;Num)}{Max(Avg.\;Cited\;Num)-min(Avg.\;Cited\;Num)}$	The capability of affection to other patent
Innovativeness	$1-\frac{Avg.\;Citng\;Num-\text{min}\left(Avg.\;Citing\;Num\right)}{Max\left(Avg.\;Citing\;Num\right)-min\left(Avg.\;Citing\;Num\right)}$	The degree of affected by other patents
Authorization	$\frac{Avg.\;Claim\;Num-min(Avg.\;Claim\;Num)}{Max(Avg.\;Claim\;Num)-min(Avg.\;Claim\;Num)}$	The scope of legal right
Marketability	$\frac{Avg.\;Family\;Num.-min(Avg.\;Family\;Num)}{Max(Average.\;Fmaily\;\#)-min(Average.\;Fmaily\;\#)}$	The scope of asserting rights

#### Module 3: Analyzing technology opportunity

The needs of the user are classified into four types by the property of needs and their related technology ability. There are two criteria to classify technology opportunity, which are the sentimental value of needs, and the ability of related technology. Table 3 shows the four types of needs based on sentimental value and technology ability.

**Table 3:** Four types of needs.

Type	Sentimental Value	Technology Ability	Technology Opportunity
1	Positive	+	
2		-	
3	Negative	+	Resolving Contradiction
4		-	Vacant Technology

In the case of type 1 and 2, the needs of users are satisfied. Thus, these types of needs can be used for a marketing strategy or a low-cost strategy to gain more consumers. These strategies are dependent on the environment of each company or developer. However, this research discovers technology opportunity from needs based on user opinions and the properties of technology. The patent index used in this research, such as sentimental value, technology ability, and keywords, cannot consider the properties and strategies of companies or developers. Thus, technology opportunities of need type 1 and 2 could not be formulated. In the case of type 3, although the ability of technology is higher than others, users are not satisfied with the satisfaction level of their needs. This means there are some gaps between the needs and related technology. Thus, this type of need can be defined as technology opportunity for resolving the gap. The gap can be analyzed from the opinion trigger and the structure of needs. Finally, in type 4, the needs of users are not satisfied, and the ability of their technology is relatively low. This means there is no specified technology for the needs of users. Thus, this case can be defined as the technology opportunity for vacant technology. Their vacancy can be analyzed from the difference between opinion triggers in user opinion and technology ecology.

Technology strategies of type 3 and 4 should be implemented, in order to resolve the contradiction, and develop the vacant technology. Detailed strategy about technology opportunity is suggested using their opinion trigger, patent keywords, and other keywords. Type 3 opportunities to resolve contradiction (RC) can be suggested based on the gap between the structure of needs and related technology. Needs that are associated with a specific function, and related technology that focuses on an object can contradict one another. In this case, a technology strategy to resolve the contradiction can be suggested using opinion triggers. Type 4 opportunities to develop vacant technology (VT) can be proposed using an opinion trigger. Based on an opinion trigger, which function of an object should be developed to satisfy users can be suggested. In addition, the sentimental value of each need and the detailed technology property of each technology are used as the source of strategy.

## Case study

This research concentrates on the car industry to extract opinion triggers, and discover technology opportunity. Since the car industry is one of the most active areas in which users express their opinion in the web, many opinion mining studies target this industry to extract user opinion [25]. The car industry has various types of users, and their needs vary, depending on the purpose. Therefore, in order to interpret their needs accurately, needs should have to be analyzed in what they want, and how it works.

### Module 1: Identifying opinion triggers

#### Dataset

Review data is collected in four websites, which are ‘amazon.com’, ‘cars.com’, ‘car and driver (www.caranddriver.com)’ and ‘the car connection (www.thecarconnection.com)’. The ‘amazon.com’ has the biggest customers and products among internet shopping malls and other websites are nominated in J.D. Power top 10 car review sites. During 2007–2016, 38,748 reviews are collected without limiting the specific model. Data fields include review title (year, make, and model), review year, review body and type of data source. Using Rselenium, the html sources from review pages are automatically crawled. Then, the html sources are partitioned to collect review data through rvest’ and ‘httr’ package. Data that does not include the review body or has a length of 10 or less in words is excluded for the analysis of word2vec and clustering. The final dataset consists of 37,697 reviews (32,260 from Amazon.com, 1,452 from Car and driver, 2,881 from Car connection, and 1,104 from Car.com).

#### Extracting opinion triggers

Prior to the case study for the suggested opinion trigger structuring, the information on raw data needs to be described. Table 4 shows the result of keyword frequency in the review data after eliminating stop-words such as ‘a’, ‘the’ and ‘and’. Consequently, keywords such as ‘car’, ‘great’ and ‘like’ mostly appeared in reviews. In addition, Table 5 shows top 5 frequency-based needs by manually interpreting user’s opinion including one of the top keywords (fuel, engine, mileage and so on), accounting for over 80% of the entire keyword frequency, as user’s needs. The needs ratio indicates the percentage of review document corresponding to each frequency-based. The extracted keywords have a limitation when it comes to analyzing user needs for technology opportunity. They hardly reflect how people satisfy/dissatisfy, which object or function it is and so on. Thus, this paper suggests novel methods to structure users’ needs as an opinion trigger by using the combination of existing techniques which cannot be analyzed through keyword frequencies.

**Table 4:** Keyword frequency in review data.

Rank	Word	Frequency	Rank	Word	Frequency
1	car	42,746	6	good	14,575
2	great	19,079	7	rear	13,878
3	like	17,612	8	engine	13,676
4	new	17,141	9	love	13,540
5	drive	16,845	10	power	12,608

**Table 5:** Needs ratio based on keywords and documents.

Rank	Needs (manually interpreted)	keyword list	Ratio
1	Engine—fuel efficiency	Engine, mpd (miles per day), fuel, liter, full, …	25%
2	Engine power	Engine, power, performance, speed, …	18%
3	Design	Design, interior, wheel, seat, pillar, …	13%
4	Driving—handling	Driving, handling, downforce, drag, …	8%
5	Suspension	Suspension, steering, stable, …	7%

Collected reviews are partitioned into 228,900 sentences, and used to extract opinion triggers. Using POS-tagging, POSs are tagged to each sentence. 2,860 words, which are formed in adverbs and adjectives, are selected as the candidates of sentimental keywords. Among the candidates, sentimental keywords with frequency in the review of higher than 100 are selected. According to the accuracy of NLP, a proper noun can be tagged as an adverb or adjective. Furthermore, some tagged words are wrong. To resolve these problems, words that are tagged as adjective or adverb more than 100 times are selected as accurately tagged candidates. Selected sentimental keywords are sorted into global sentimental keyword and static sentimental keyword by their meaning. Table 6 shows 424 selected sentimental keywords and their type.

**Table 6:** Extracted sentimental keywords from opinions.

	Positive		Negative	
	The number of words	Sentimental keyword	The number of words	Sentimental keyword
Global	40	good, top, best, great, excellent …	22	bad, poor, odd, oddly …
Static	278	powerful, quick, easy, fast, huge …	84	silly, mushy, awkward, dull …

Review sentences are divided into opinion sentences (OS) and non-opinion sentences (NOS), to identify opinion triggers based on sentimental keywords. In all, 115,277 sentences are sorted into OS, while 113,623 sentences are sorted into NOS. Using the word frequency of each sentence and Naïve Bayes classifier, the appearance probability of each word in an opinion sentence can be calculated. Based on this probability, 368 opinion triggers are identified. In addition, in order to analyze an opinion trigger’s property in the structure of needs, each word’s POS is tagged. Then, an opinion trigger formed as a verb is sorted in the function opinion trigger. In addition, an opinion trigger formed as a noun is sorted in the object opinion trigger. Table 7 shows each opinion triggered type and probability.

**Table 7:** Identified opinion triggers (part).

Word	POS	Type	Occurrence		P(Word|OS)	P(Word|NOS)
			OS	NOS*		
seat	Noun	Object	5,782	4,856	0.006425	0.004874
power	Noun	Object	5,152	5,184	0.005725	0.005203
ride	Verb	Function	4,637	2,322	0.005153	0.00233
interior	Noun	Object	4,548	3,022	0.005054	0.003033
steer	Verb	Function	2,971	2,655	0.003302	0.002665
space	Noun	Object	2,552	2,232	0.002836	0.00224
fuel	Noun	Object	2,487	2,000	0.002764	0.002007
accelerate	Verb	Function	2,474	1,110	0.002749	0.000111
design	Noun	Object	2,458	2,133	0.002732	0.002141
price	Noun	Object	2,420	2,451	0.002689	0.00246
gas	Noun	Object	2,199	1,236	0.002444	0.001241
handle	Noun	Object	2,190	1,193	0.002434	0.001197

### Module 2: Analyzing the needs of user & related technology

Patents are collected from wisdomain.com, which provides search and download services for registering United States patents and the trademark office. Using queries structured by car and related to car, such as vehicle, automobile, and automotive, 63,358 patents that are registered in 2007 through 2016 are collected. Based on the Word2Vec algorithm, the title, abstract, and claim in patents and user reviews are embedded in a two-dimensional vector space. In Word2Vec, 1,500 words that occur more than 100 times are used. The window size that interprets contextual relationship with each other is set as 4. Then, 200,000 times repeated training is conducted. To analyze the relationship and contextual similarity between each word, PAM is conducted to make 308 clusters, the same as the number of triggers. After clustering, the contents of clusters are interpreted by opinion triggers, sentimental keyword, and normal keywords.

Then, 51 clusters containing at least one trigger in the cluster are interpreted as the needs of the user. Among the derived needs, 22 needs that could not be satisfied by technology development, such as design and price, are excluded. Since this paper focuses on technology-related user need that can be handled with R&D activities, opinions distant to technology problem such as ‘satisfaction of moon-roof style and design’ are eliminated manually. Table 8 shows 6 representative needs of users among 51 total needs.

**Table 8:** Discovered needs of users (part).

Trigger	Type	Needs
cargo, transport, move	OOT+FOT	Cargo Transportation
downforce	FOT	Downforce of car
squeal, shift, squeak	FOT	Noise in brake system
moves, roadholding, roadhold	FOT	Road holding of car
cushions, control, handling, steer, handle, understeer	OOT+FOT	Control using handle
passengers, safety travels, security	OOT	Stability of passenger

Sentimental keywords are used to extract the sentimental value of each need that is targeted to discover technology opportunity. Based on the graph-based semi-supervised learning, the sentimental values of needs are calculated using sentimental keywords and opinion triggers. The sentimental value of 424 sentimental keywords is calculated in Semantria. Their sentimental values are propagated in different ways, depending on the sentimental keywords type. Table 9 shows the sentimental value of candidate needs for technology opportunity; 65 percent of needs are positive, while 35 percent of needs are negative.

**Table 9:** Sentimental value of needs for candidate technology opportunity.

Needs no.	Sentimental Value	Needs no.	Sentimental Value
1	-1.0788	16	-4.8443
2	7.0773	17	5.9294
3	-5.3899	18	11.6370
4	15.5756	19	6.0553
5	-7.7238	20	6.9009
6	-1.8141	21	7.6814
7	3.3149	22	1.0182
8	3.1905	23	2.9164
9	-1.2658	24	-15.2555
10	17.9932	25	4.6036
11	6.8940	26	8.4965
12	-3.8543	27	2.1826
13	4.0034	28	-0.8839
14	3.3888	29	-1.6937
15	1.8362		

To identify the relationship between needs and technology, patents are allocated based on their keywords type. If needs are structured with function and object opinion trigger, patents that have object keywords or function keywords are allocated in needs. If needs are structured with one type of trigger, patents that have the same type of keyword are allocated in needs. Then, the technology ability is analyzed by calculating the number of cited/citing patents, the number of INPADOC family countries, and the number of independent claims. Table 10 shows the relationship between needs and their normalized technology ability in relative value.

**Table 10:** Needs-technology relationship and technology ability.

Needs No.	Structure	Patent ratio		Technology Ability	Needs No.	Structure OOT	Patent ratio		Technology Ability
		OOT	FOT				FOT	FOT	
1	OOT+FOT	0.0367	0.1230	0.2067	16	OOT+FOT	0.1142	0.0539	-0.0012
2	FOT		0.0008	0.0322	17	OOT+FOT	0.2142	0.1971	-0.0053
3	FOT		0.0445	0.0806	18	FOT		0.2706	-0.0091
4	FOT		0.0045	0.0135	19	OOT+FOT	0.0946	0.0246	-0.0075
5	OOT+FOT	0.0417	0.0086	-0.0207	20	OOT	0.0408		-0.0715
6	OOT+FOT	0.0242	0.0027	-0.0316	21	OOT	0.1315		0.0174
7	OOT	0.6203		-0.0892	22	OOT	0.1101		-0.1013
8	FOT		0.0116	0.0529	23	OOT	0.6191		-0.0665
9	OOT+FOT	0.1857	0.0026	-0.0210	24	OOT+FOT	0.3391	0.0138	-0.0966
10	OOT+FOT	0.0626	1.0000	0.0086	25	OOT+FOT	0.2029	0.0000	0.0415
11	OOT+FOT	0.5712	0.1822	0.0554	26	OOT	0.3343		-0.0284
12	OOT	0.3969		0.0102	27	OOT	0.0000		-0.0529
13	OOT	0.1348		-0.0771	28	OOT	0.0239		-0.0110
14	OOT+FOT	0.2152	0.3225	0.0062	29	FOT		0.1187	0.0908
15	OOT+FOT	0.0276	0.2476	-0.1007					

### Module 3: Discovering technology opportunity

Using the sentimental value of needs and technology ability, all identified needs are classified into four types, as in Table 11. Type 1 needs have positive user opinion and high technology ability. These are related to the driving environment and core hardware, such as road-holding, acceleration ability, suspension, and acceleration pedals. Since their ability can be expressed by an objective index, the results could be interpreted by high technology ability derived high user satisfaction. Type 2 needs have positive user opinion; however, their technology ability is low. These are related to passenger or driver feeling, such as being comfortable while driving, hardware manipulation system, fuel efficiency, safety driving, and backseat and back of the seat. Driver or passenger feeling could not be calculated by quantitative value, and personal preferences are too diverse to calculate one criterion. Thus, although their technology ability is low, the user feels satisfaction about the needs. Type 1 and 2 needs have to be interpreted by product or company property, such as marketing property, development strategy, and industrial environment. Though various indices used in this study, such as sentimental value, opinion trigger, and needs–technology relationship, they cannot reflect their property related to marketing property or industrial environment. Thus, this research focuses on type 3 and 4, which can be resolved by investigating the contradiction type and vacant technology type. Table 12 shows the target of this research to formulate a technology strategy.

**Table 11:** Classified needs and technology opportunity.

Type	Sentimental Value	Technology ability	Technology Opportunity	The number of needs(ratio)
1	Positive	+		8(27%)
2		-		11(38%)
3	Negative	+	Resolving Contradiction	4(14%)
4		-	Vacant Technology	6(21%)

**Table 12:** Property of technology opportunity.

No	Index	Trigger type	Sentimental Value	Technology Ability	Object Patent Ratio	Function Patent Ratio	Technology Opportunity
1	Cargo Transportation	OOT+FOT	-1.0788	0.2067	0.0367	0.1230	RC*
2	Noise in brake system	FOT	-5.3899	0.0806		0.0445	RC
3	Control using handle	OOT+FOT	-7.7238	-0.0207	0.0417	0.0086	VT**
4	Warning for battery	OOT+FOT	-1.8141	-0.0316	0.0242	0.0027	VT
5	Seat adjusting	OOT+FOT	-1.2658	-0.0210	0.1857	0.0026	VT
6	Audio	OOT	-3.8543	0.0102	0.3969		RC
7	Downshift	OOT+FOT	-4.8443	-0.0012	0.1142	0.0539	VT
8	Tablet mounted in car	OOT+FOT	-15.2555	-0.0966	0.3391	0.0138	VT
9	Navigation	OOT	-0.8839	-0.0110	0.0239		VT
10	Noise in charging for electric car	FOT	-1.6937	0.0908		0.1187	RC

The technology opportunity (Type 3) for resolving contradiction has high technology ability, but users are not satisfied with their needs. In other words, their technology properties cannot satisfy user needs. Need #1, for ‘cargo transportation’ has a high relationship with transportation, which is a functional opinion trigger. But in the user opinion, there are many keywords related to safety, such as stable, parallel, and horizontal. Thus, transportation technology that aims to improve cargo safety has to be developed. Need #2, the ‘noise using brake’ is constructed by functional opinion trigger. There are many keywords related to rapid brake systems, such as immediate, response, and deliver. On the other hand, there are no keywords related to noise that occurs in opinion. That is, a brake system that aims to facilitate rapid operation cannot satisfy user needs for a quiet brake system. To satisfy the needs of users, the brake system has to develop a quieter operating method. Need #6, for ‘audio’, is constructed by object opinion trigger. Although technology related to the need is just installed in the speaker or audio in a car, it does not have special characteristics for the car. In particular, a user wants a great sub-woofer. However, few patents tackle the sub-woofer part. Thus, audio (speaker) for a car should have to focus on a superior sub-woofer and specialized property for a car. Need #11, concerning ‘charging noise for electric vehicle’ is constructed by function opinion trigger. Patent keywords in the cluster are related not to sounds and noise, but to efficiency. On the other hand, keywords from opinions said that the user wants a silent charging system. Thus, noise-free charging technology, or another technology that can reduce noise from charging, should be developed.

Technology opportunity for vacant technology means they do not have the needs-related technology, or low technology ability fails to satisfy the needs of the user. This type (Type 4) of need should have new technology developed, or be as specialized as the user wants. Need #3, concerning ‘control using handle’ has the related technology. However, their technology ability is low. Furthermore, they do not have keywords related to sensitivity. Since the notion of more sensitive and sophisticated control systems is derived from user opinion, more accurate vehicle handling technology needs to be developed. Need #4, ‘warning for battery’ has various patents related to battery, power, warning, and alarm. However, although each keyword is included in different patents, the combination of keywords that is relevant to user needs does not occur in a single patent. In addition, the ability of each technology is low. Thus, a technology that can warn of the remaining level of battery has to be developed. Need #5, about ‘adjusting seat’ has many patents that are related to the seat. However, there are few patents related to adjusting. In addition, automatic and personalization occur in user opinions. Consequently, car seat technology has to be developed to enhance the adjusting function, and automatic and personalization option. Need #7, about ‘downshift’ is related to a gear that is the object of need. However, downshift that is in the gear functional side is not emphasized in the patent. Therefore, downshift technology has to be developed, aiming at the function itself. Need #8, about ‘tablet mounted in car’ has many patents related to a tablet that is the object of needs. However, touch or recognition-related patents that are functions of need are relatively small, and their technology ability is low. Thus, tablet-related patents focusing on touch and recognition have to be developed. Need #9, ‘navigation, does not have specialized keywords. Therefore, a technology strategy could not be formulated.

### Verification

At first, this research compares the proposed approach with conventional methods to show the validity of the main methodologies based on word2vec and PAM. Identical data that was used in the proposed approach is analyzed by using Document Term Matrix (DTM) and k-means clustering algorithm, which both are frequently used in text mining and clustering. DTM is used to text mining analysis such as Latent Semantic Analysis (LSA) and Latent Dirichlet allocation (LDA) and has a limitation of a frequency-based approach. K-means clustering is also one of the traditionally popular clustering methods. Table 13 shows the list of keywords in five groups of clusters which 308 clusters derived by DTM and k-means clustering algorithm are assigned into. The result that the ‘brand’ cluster type includes most of the clusters (176 clusters) shows that most people who wrote down their own opinion on the website mentioned a particular brand name. Thus, the result is subject to provide insignificant information on customer needs. In addition, document clustering has the limitation that it can rarely derive detailed and concrete needs because there are various opinions in one review document. In these reasons, this paper has a contribution that can define segmented needs by starting from an ‘opinion trigger’ and structuring opinion text data.

**Table 13:** Cluster type based on DTM & K-means clustering.

Rank	Cluster types	List of representative keywords in each cluster	Number of clusters
1	Brand	Kia, Optima, Tesla, Volvo, s60, audi, honda, lexus …	176
2	Engine	Engine, power, performance, mpd, speed, …	29
3	Component	Rear, front, door, pillar, latch, …	6
4	Sentiments	Good, like, love, great, luxury, best, worst, bad, …	3
5	Car type	Truck, sedan, electronic, hybrid, coupe,…	4

In addition, the main results of discovering user needs are compared with reliable and actual data. The complaints data provided by Office of Defects Investigation (ODI) of National Highway Traffic Safety Administration (NHTSA), which is a part of the Department of Transportation of the Executive Branch of the U.S. government is employed for the comparison. The ODI complaints data is utilized to help make decisions of recall or other safety-related problems, containing over than 1.5 million safety-related defect complaints received by NHTSA since January 1, 1995 (https://www-odi.nhtsa.dot.gov/downloads/). Among all fields of data, the ‘description of the complaint’ is analyzed to derive the main issues that users have reported dangerous and problematic when they use a car. Although the description is revised by a recorder and mainly focuses on safety-related problems, the ODI complaints data is suitable to convince the identified needs for verification. Table 14 shows the frequency and example of ODI complaints data related to the identified user needs. The needs of ‘control using handle’ has the highest record in ODI complains 94,168 times and the complaints linked to the needs accounts for around 10% of total ODI complaints. It reveals that the needs extracted by the proposed approach reflect consumers’ actual discontents and opinions.

**Table 14:** ODI complaints data related to the needs.

Needs	ODI Complaints data related to the needs	
	Freq.	Example of description
Cargo Transportation	2	When **transporting cargo** behind rear seats, if cargo hits back of seats, seats fly forward, happens when suddenly applying brakes, dealer readjusted latches, snapped latches do not hold.
Noise in brake system	7,798	**Brake noise**.
Control using handle	94,168	**Possible steering failure**, vehicle swerved from side to side out of control causing injury to consumer, consumer also suspects possible firestone tire failure could have caused loss of control.
Warning for battery	170	**Vehicle shut off without prior warning. Battery/ alarm system/** key, and starter were checked.
Seat adjusting	1,711	**Seat adjustment controls too close together, causing accident** when drivers seat back fell when driver was trying to move seat forward
Audio	1,140	Front sway bar bushing malfunctioned. In addition, rear u-joints/ air conditioner condensor/door panels/hood latch and **right front speaker failed**.
Downshift	2,381	Check engine light keeps going off and on sometimes loosing legal driver speed. **Then must downshift of press all the way down on the gas pedal until it reaches normal speed**.
Tablet mounted in car	6,735	Power door locks failed; also electrical **display functions module failure**/ front end misalignment/engine noise/fuel pump noise/ leaf spring failure and replaced a/c fan motor.
Navigation	729	Steering mechanism on the vehicle would lock up at the end of the steering rotation. **navigational system**, radio, bumper and windows were defective.
Noise in charging for electric car	1	Sometimes when the heater was running, i heard a pop noise from left front area of the car. Without warning or engine lights. **While charging up electrical battery vehicle caught on fire**.
Total	114,835	(10% of total ODI Complaints data is related the suggested user needs)

The accuracy of machine learning techniques has been verified through repetitive analyses of 100 times in order to show the consistency of the analysis results. Table 15 illustrates the accuracy of conducting word2vec and PAM for 10 needs that were identified from the ODI complaints data. The hyper-parameters such as ‘skip_window’, ‘batch size’ and ‘embedding size’ of the machine learning technique need to be defined by conducting experiments to make combined words like Benz* (Benz C-class) be closely positioned in a word map. In this paper, consequently, the values of ‘skip_window’, ‘batch size’ and ‘embedding size’ become 8, 128 and 64, respectively after the repetitive experiments. On average, the learning accuracy on the 10 needs is 78.1%, which means that the 10 needs are consistently derived in 78.1 times out of 100 tests on average. Five needs (Noise in brake system, Control using handle, Audio, Downshift, Noise in charging for electric car) among the 10 needs are perfectly extracted on every trial by the proposed approach. On the other hand, warning for battery (7%) and cargo transportation (18%) show low accuracy. However, such needs that have low accuracy can be interpreted as new needs. The suggested machine learning algorithm reflects both pre-defined needs for technology opportunities and newly derived users’ needs, reflecting the objectives of this paper to explore potential users’ needs. The results of the verification will be discussed in the section of discussion.

**Table 15:** Machine learning (Word2vec + PAM clustering) accuracy.

Needs		Accuracy	Newly defined needs
1	Cargo transportation	18%	Cargo space, Cargo seatback, Damp transport
2	Noise in brake system	100%	-
3	Control using handle	100%	-
4	Warning for battery	7%	Electric battery knockoff, Battery active safety
5	Seat adjusting	97%	-
6	Audio	100%	-
7	Downshift	100%	-
8	Tablet mounted in car	96%	-
9	Navigation (direction)	63%	Navigation infotainment, (Goodlooking navigation equipment)
10	Noise in charging for electric car	100%	-
Total average		78%	

In this research, technology opportunities are derived from the needs of the user. To verify the opportunities, two-step verification is conducted. First, the needs of the user are verified by their persistence. Since technology development for the temporary needs of users brings small profit, the persistence of needs should be verified before R&D planning. Second, technology opportunities are verified by interests. If many competitors have interests in the same technology, this can promote a highly competitive environment. Thus, a developed technology cannot survive in this environment and gain competitive advantage. First, needs that users continue to express their opinion to others can be interpreted as a criterion for their choice. Thus, persistent needs should have to be satisfied by developing specialized technology. To compare the persistence of needs, Table 16 shows the average needs growth rate from 2009 to 2016, and 2016 to 2017. Needs # 2, 3, 7, 8, 9 have a higher growth rate from 2016 to 2017, than from 2009 to 2016. This means these needs have to be satisfied to get user choice and competitive advantage.

**Table 16:** Annual growth rate of needs.

Needs No.	Annual needs growth rate	
	2009~2016	2016~2017
1	0.1087	-0.0942
2	0.3635	0.8125
3	0.3883	1.1200
4	0.1011	-0.2355
5	0.1113	-0.2582
6	0.1445	-0.5455
7	0.1801	0.6508
8	0.0792	0.8000
9	0.3760	0.7500
10	0.9035	0.4286

Technology interest can be analyzed by the patent registration rate. In this research, whole technology fields patent growth rates and needs-related technology growth rates are compared. If the needs-related technology growth rate is low, this means that other competitors do not have interests in satisfying the needs of the user. In other words, needs-oriented technology can easily be chosen by the user. Because there is no technology, it can satisfy their needs. To show the technology interests, Table 17 shows the annual patent growth rate of needs-related technology. The annual patent growth rate of the whole technology is analyzed as 9.33%, while the patent growth rate of needs # 2, 5, 6, 7, 8 and 9 are lower than 9.33%. In addition, their growth rate ranks in 29 valid relationships between needs and technology are low. This means these needs have low interest in car technology. That is, needs-oriented technology gets more choices from users and high competitive advantage.

**Table 17:** Annual patent growth rate of needs.

Needs No.	Annual patent growth rate	rank
1	14.99%	4
2	7.47%	27
3	11.67%	9
4	12.78%	8
5	8.19%	22
6	6.61%	30
7	8.06%	23
8	8.32%	18
9	8.21%	21
10	11.26%	11

## Discussion

This paper has proposed a novel opinion mining methodology to structure user needs through the concept of Opinion Trigger focusing on objects and functions in user review data in the automotive area as Table 8. User needs have been derived through the proposed methodology and explored the technology opportunities based on the needs as Tables 12 and 13. Also, the result of the study was verified using comparison with time interval, ODI complaint data which shows ground truth, and the accuracy of machine learning methods as Tables 14, 15, 16 and 17. Based on these results, this study discusses in a view of comparison with related studies and actual/current issues in the automotive filed.

At first, the accuracy of machine learning is discussed based on Table 15. The accuracy of the study was verified in terms of the consistency of the consecutive analysis results of word2vec and PAM clustering. The 10 user needs derived from the study have a large range of accuracy according to each need. Needs with high accuracy are generally concerned with the purchase of a car or with a complaint of actual use. Indeed, needs with high accuracy of 90% or more tend to occupy high frequency of 1,000 or more data in ODI complains data including ‘noise in brake system’, ‘control using handle’, ‘seat adjusting’, ‘audio’, ‘downshift’, and ‘tablet mounted in car’. Handle control and noise in brake system have had a big issue in the real industry such as recall, and the accuracy of needs related to this problem is very high. Those needs are dealt with previous related study. ODI and amazon review data are analyzed and indicated that the issues on power steering failure on Cobalt 2006 and rear brake issues on Honda Accord 2008 are directly related to the results of topic modeling of online review data [49, 50]. In addition, the users’ need of power steering failure is shown as one the main results using topic modeling for review data on specific car model [51]. On the other hand, needs with low accuracy including cargo transportation, warning for battery and navigation (direction) can be interpreted as potential needs, which are not yet manifested as general needs such like high accuracy needs. In this study, word embedding vector considers relations between similar words before clustering for extracting user needs. Based on vector similarity, potential needs are derived by considering potential inter-word connectivity. Thus, although the frequency is low and does not appear with high accuracy in 100 times of test analyses, new needs can be derived from a potential perspective. ‘Cargo transportation’ and ‘Seat adjusting’ have been an issue in the actual automotive industry, which is verified by an industry player who is one of domain experts in Hyundai motor group. Cargo transportation needs can be interpreted as significant needs because the general user is interested in a freight transport (such as outdoor items) along with the increase in purchases of SUVs and CUV vehicles [52, 53]. The need on battery can be described that many companies are currently competing to gain technological advantage such as hybrid, full hybrid and plugged-in hybrid. In other words, low-accuracy needs reflect the needs of users who have recently become an issue, which has never been derived from previous studies.

Moreover, this study has compared the growth rate by dividing into the last 2 years and the previous years in the total data collection period, as shown in Table 16. In the case of the need of audio, which decreases most from the past, it can be interpreted that the high-performance technology has already been completed or that the user has reached its limit utility [54, 55]. On the other hand, the need of control using handle can be interpreted as an increase in the variety of automobile types such as sports cars and SUVs, as well as the increasing interest in handling an advanced driving assistance system (ADAS) among the automobile options that have recently become an issue [56, 57].

## Conclusion

The study proposes a novel concept of ‘opinion trigger’ to extract user's needs from review data, in order to structure unstructured review data for technology opportunity discovery. This research defines an opinion trigger as an object towards which users directly express their needs. Based on the opinion trigger, the needs of the user are analyzed into object and function, rather than positive or negative feelings about a product. The object opinion trigger and function opinion trigger could be interpreted as what they want, and how it works, respectively. In addition, the word gap between users and technicians could be removed, and related technology can be derived without any additional process, through using the opinion trigger and Word2vec algorithm. Using the needs and the related technology index, two technology opportunities are derived. The first opportunity has resolved the contradiction between technology ability and sentimental value. Using technology-related ratios and opinion triggers, the reason for contradiction can be analyzed. The second opportunity is the vacant technology that has low technology ability to satisfy the needs of the user. The needs of the user were investigated through the opinion triggers, sentimental keywords, and keywords from opinions. Thus, a technology strategy for the two types of opportunities was suggested. Finally, the suggested strategy was verified by the insight on the increasing ratio of needs and technology competitors.

This research has several theoretical contributions. First, a feature of word2vec that words with high semantic similarity appear in adjacent vector space is used as a clustering methodology. Since previous studies have focused on just the vector source of each word, they use vector calculation or distance between words to interpret a single word. However, this research interprets the needs of users through a combination of words using the clustering methodology. Second, the proposed methodology uses linguistic information based on natural language processing. Though previous text mining methodology uses the meaning of words and their frequency, this methodology can bring a multi-dimensional text mining approach. Using opinion mining, the sentimental value is propagated to neutral words in the existing methodology. In addition, single words are structured by user needs, using their relationship and part of speech. The suggested methodology using both word2vec and K-medoid clustering has a contribution point that extracts potential information by considering the inter-word relationship based on word embedded vector unlike traditional topic modeling of LDA, which simply have a statistical approach to documents and words.

This research can produce efficiency in R&D management and industry fields in various ways. First, the suggested systematic approach can explore the user’s needs that are freely presented by the user and updated in real time without the large-scale survey for user satisfaction, which needs a lot of time and money. The main reasons for user opinions can be extracted, and a strategy made to increase users satisfaction with their products. From the case study of the car industry in this research, user needs for a speaker and navigation module are extracted. The speaker and navigation are not the main components of the car. However, users actively express their opinions about the speaker and navigation. In other words, even if the needs of the user are not the main component of their industry, they can be analyzed through the proposed methodology. Second, there is no additional process to reflect user opinions in the R&D process. The minimized R&D process can generate more economic profits, and make the R&D management process more flexible. The proposed methodology can present the structured needs result through the concept of opinion trigger structured by object and function, and it can utilize the result itself without additional work of analysis result unlike existing topic modeling. Third, a contradiction that high technology ability sometimes does not bring users high satisfaction can be resolved. Based on an opinion trigger, the main reason for user opinion and related technology can be clearly identified. By analyzing the relationship between needs and technology, the reason why technology cannot satisfy the needs of the user is suggested. An effective way to satisfy the needs of the user by developing exact technology attributes is suggested by analyzing those reasons.

However, this research has several limitations. First, one trigger can derive only one need. For example, an opinion trigger ‘design’ could be interpreted by ‘window design’, ‘roof design’, and ‘tire design’. However, since the PAM allows one keyword to one cluster, one trigger cannot form various needs. Second, the suggested methodology discovers technology opportunities in a specific technology. Various technology innovation insights, such as heterogeneous technology fields or technology fusion, cannot be reflected in the opportunities. To resolve these limitations, a new clustering methodology, such as overlapping k-means clustering, can be adopted for the suggested approach. Since the overlapping k-means clustering can assign one trigger to various clusters, it can derive more reasonable results. In addition, patents in more various technology fields should be collected, to reflect various technology innovation ideas.
